# The effect of pharmaceutical supply chain management practice on operational performance: the case of the central clusters and central Ethiopian pharmaceutical supply service

**DOI:** 10.1186/s40780-025-00533-3

**Published:** 2026-01-03

**Authors:** Bekele Boche, Temesgen Dinsa, Zemenu Wube Bayleyegn, Azmeraw Bekele

**Affiliations:** 1 Department of Social and Administrative Pharmacy, School of Pharmacy, Institute of Health Sciences, Jimma University, Oromia, Ethiopia; 2 Department of Pharmacy, College of Medical and Health Science, Wollega University, Nekemte, Oromia, Ethiopia; 3 Department of Social and Administrative Pharmacy, School of Pharmacy, College of Medicine and Health Sciences, University of Gondar, Gondar, Ethiopia

## Abstract

**Background:**

Effective pharmaceutical supply chain management ensures the availability of medicines and improves healthcare, while supply chain integration and transparent sharing of quality information are critical to increasing profitability and performance. However, the Ethiopian pharmaceutical supply service faces persistent gaps that impede operational performance. Thus, this study aimed to examine the effect of supply chain management practices on the operational performance of the central clusters and the central Ethiopian pharmaceutical supply service.

**Method:**

A cross-sectional study was conducted from May to July 2024. The study included all employees from central Ethiopia’s pharmaceutical supply services and central clusters, who were primarily engaged in pharmaceutical supply chain management practices. Thus, a census of supply chain management-practicing staff enabled a thorough examination of operational processes within the target organizational units. Data were collected using five-point Likert scale questionnaires adapted from the literature. Descriptive and inferential statistics, including multiple linear regressions, were employed to determine the relationships between supply chain management practices and the operational performance of the Ethiopian pharmaceutical supply service.

**Results:**

The results indicated that quality is a primary criterion in supplier selection (mean = 3.34) and that there is a tendency to rely on a few high-quality, committed suppliers. Information sharing practices were moderate, with 46.2% agreeing that proprietary data is shared and 50.3% confirming transparent communication from trade partners (mean = 3.51). Moreover, the results indicate a moderate to strong positive linear relationship (*R* = 0.576) between the predictors and the outcome, with a statistically significant correlation that explains approximately 33.2% of the variance in the outcome. Strategic supplier partnerships (β = 0.168, *p* < 0.010), the level of information sharing (β = 0.165, *p* < 0.006), the quality of information sharing (β = 0.126, *p* < 0.042), and internal integration practices (β = 0.224, *p* < 0.030) had positive and significant effect on operational performance of central Ethiopia’s pharmaceutical supply services and central clusters.

**Conclusion:**

Ethiopia’s pharmaceutical supply services show moderate to strong performance, with areas needing improvement. Customer service and operational performance were positive, though gaps exist in customer satisfaction evaluation. The quality of information sharing, strategic supplier partnerships, effective information sharing, and internal integration practices collectively drove effectiveness, explaining a substantial portion of performance variability.

## Background

 Supply chain management (SCM) plays a critical role in healthcare systems, encompassing the efficient and coordinated transfer of pharmaceuticals and services from suppliers to end-users [1, 2]. This requires the seamless integration of logistics, strategic coordination with manufacturers, suppliers, and distributors, as well as interdisciplinary collaboration across procurement, sales, finance, and information systems [3, 4]. Effective supply chain integration and transparent information sharing between buyers and vendors are important strategies for enhancing overall supply chain profitability and organizational performance [5, 6]. Central to strategic supplier partnerships is information integration, which relies on the timely, accurate, and high-quality exchange of strategic, tactical, logistical, customer, and market-related data, ensuring minimal delays and distortions to enable evidence-based decision-making and optimize SCM effectiveness [7, 8]. Strategic agility serves as a partial mediator in the relationship between supply chain management activities and operational performance, while supplier alliances, customer relationship management, logistics, and information/knowledge sharing all contribute to quantifiable gains in organizational efficacy and efficiency [9, 10]. These interrelated components highlight how crucial a knowledge-driven, agile, and cooperative strategy is to maximize supply chain results and attain long-term operational excellence [7, 11]. Furthermore, internal integration is an essential SCM practice that harmonizes business information systems at the operational level, fostering structural and social collaboration to enhance organizational agility, optimize processes, and minimize waste [7, 11, 12]. Unfortunately, some companies experience inadequate supply chain practices, such as inefficient inventory management, operational wastage, poor supplier relationships, and integration issues that negatively affect operational performance, profitability, and overall business efficiency [13–15]. Ineffective supply chain management escalates costs and customer dissatisfaction, eroding trust through delays, fragmented supplier relationships, and integration challenges, ultimately impairing operational performance and organizational efficiency [16, 17]. Ethiopia’s pharmaceutical supply services (EPSS) have been facing significant challenges, such as inventory losses, stock imbalances, product shortages, weak integration, and poor information sharing, which impede the organization’s performance [11]. Inadequate internet connectivity, infrastructure gaps, a lack of skilled personnel, and frequent power outages hindered effective supply chain management in EPSS, leading to poor data quality, information-sharing challenges, and pharmaceutical shortages or wastage [18]. Another study conducted in EPSS indicated that its supply chain was inefficient; only 14.8% of orders were properly fulfilled, 37 items were out of stock for an average of 8.5 days, and $2 million was wasted [19]. Thus, this study aimed to examine the effect of pharmaceutical supply chain management practices on the operational performance of the Central Ethiopian Pharmaceutical Supply Service (CEPSS) and the central cluster, providing evidence-based insights to optimize efficiency, reduce costs, and enhance healthcare delivery.

## Theoretical framework

**Fig. 1:**
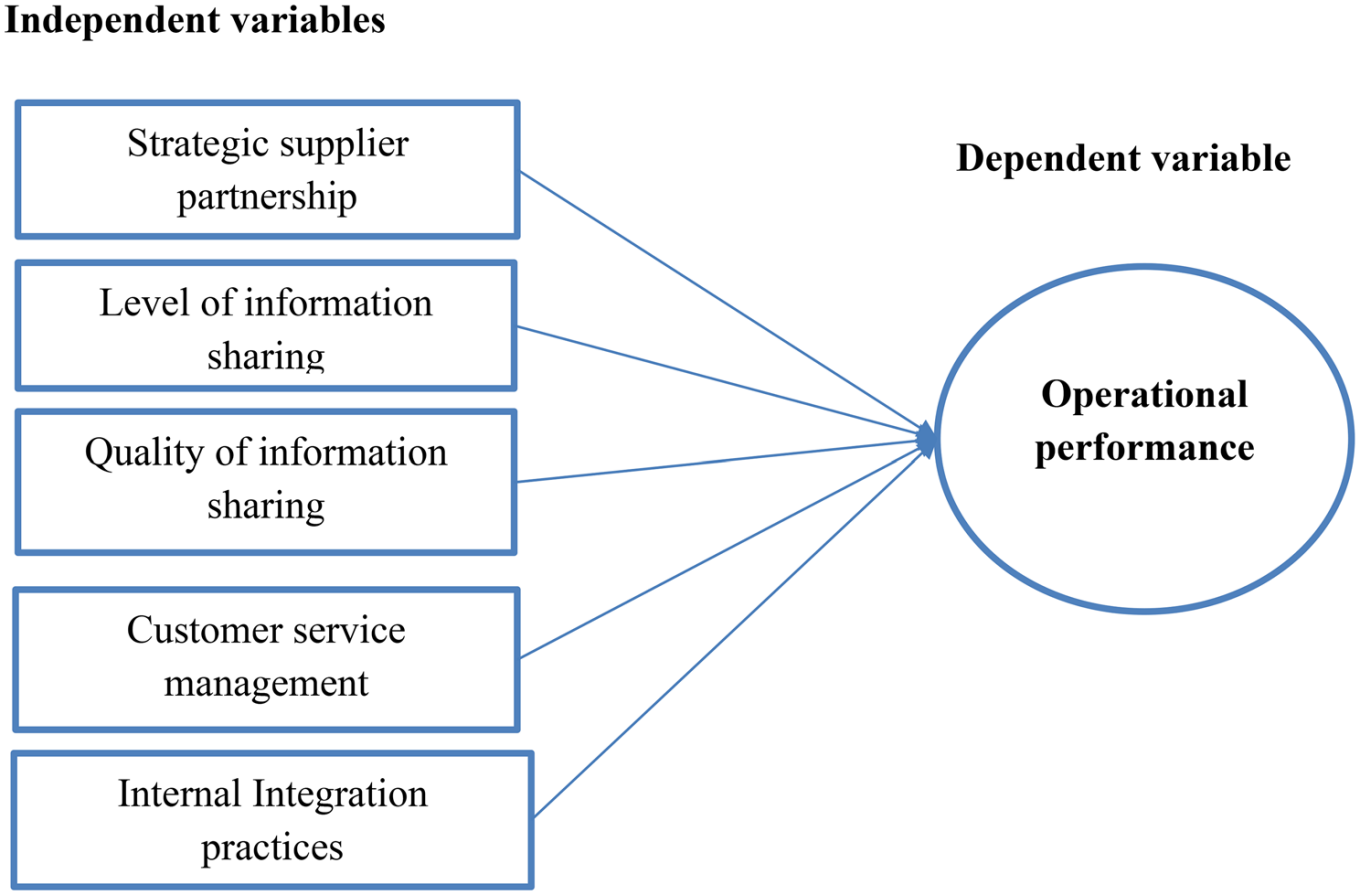
Conceptual framework of the study

The study employs the Resource-Based View (RBV), Relational View Theory, and Systems Theory to define how SCM practices affect operational performance. The RBV suggests that a company’s long-term competitive advantage originates from its unique resources and competencies, which are valued, rare, difficult to copy, and not easily substitutable [20–22]. Relational View Theory suggests that a firm’s competitive advantage can be developed not only through internal resources (as in Resource-Based View), but also through inter-organizational interactions. According to this theory, enterprises can acquire a sustainable competitive advantage through strategic alliances, collaboration, trust, information sharing, and joint investments with supply chain partners [23]. Systems theory provides a framework for understanding and managing supply networks as complex, interdependent systems. It focuses on the links and interactions between various supply chain components, including suppliers, manufacturers, distributors, and customers. Systems theory can help to optimize supply chain design, implementation, and administration by considering the impact of changes in one component on others. This holistic approach enables better decision-making, improved efficiency, and more resilience in the face of disturbances [24] (Fig. 1).

### Hypothesis

#### H1

Strategic supplier partnerships have a significant positive effect on the operational performance of CEPSS and the central clusters.

#### H2

The level of information sharing has a significant positive effect on the operational performance of CEPSS and the central clusters.

#### H3

The quality of information sharing has a significant positive effect on the operational performance of CEPSS and the central clusters.

#### H4

Customer service management has a significant positive effect on the operational performance of CEPSS and the central clusters.

#### H5

Internal integration practices have a significant positive effect on the operational performance of CEPSS and the central clusters.

## Method

### Study area, design, and period

The EPSS is the public institution that supplies pharmaceuticals throughout the country. Currently, the EPSS performs pharmaceutical quantification, procurement, warehousing, and distribution activities to supply public health facilities across the country. The 19 hubs managed under the CEPSS Addis Ababa are organized into seven clusters based on geographical proximity to enhance service delivery, management, and coordination. The central clusters are among the seven clusters, which comprise Addis Ababa 1 and 2 clusters. Addis Ababa 1 cluster serves seven sub-cities in the capital, Western Shoa, South Western Shoa, and some surrounding areas of Addis Ababa from the Oromia Region and Gurage zone, Hosaena, and two Districts of Silte zone. Addis Ababa 2 cluster, instead, serves Gulele, Bole, and Yeka sub-cities in Addis while it covers the Northern Shoa zone of the Amhara region, the Northern Shoa of the Oromia zone, and the surrounding areas of Addis Ababa [25]. This study employed a cross-sectional study design and was conducted from May to July 2024.

### Study population

The whole employees at the CEPSS and the central clusters who were in charge of pharmaceutical supply chain activities made up the study population.

### Eligibility criteria

Each of the CEPSS and the central clusters’ staff actively engaged in supply chain operations was included in the study. Staff who were unavailable during the data collection and unwilling to take part in the study were excluded from the study. This study focuses exclusively on the employees actively involved in SCM activities. Non-SCM employees were excluded as their roles do not directly impact supply chain operations.

### Sample size and sampling procedure

The CEPSS and Central cluster, including Addis Ababa 1 and 2 branches, was purposively selected in particular because it is the top pharmaceutical supply service provider that has the opportunity to negotiate with domestic and/or international suppliers. Within the CEPSS and the central clusters (*N* = 312), we found 175 employees (56.1%) whose primary roles were SCM activities. These employees included in this study represented the complete subgroup of SCM-practicing staff within the organizational framework. This complete census of SCM-practicing staff enabled a thorough examination of operational processes within the target organizational units.

### Data collection instrument and procedure

The data includes respondent demographics, related factors, and operational performance (cost, quality, delivery, and flexibility) that were adapted from works of literature [7, 11, 12, 26]. A total of 175 self-administered, structured questionnaires were distributed across various departments of the CEPSS and the central clusters. The questionnaire consisted of five-point Likert scale questions that ranged from strongly disagree to strongly agree. Two former first-degree central staff members from Addis Ababa were recruited as data collectors under the supervision of the principal investigator.

### Data processing and analysis

The data were sorted and coded, then entered into Epidata 3.1 and exported to SPSS version 26 software for analysis. Descriptive and inferential statistical analyses were conducted to summarize the findings. The descriptive findings were analyzed and presented as frequencies, means, and percentages. To interpret the mean scores of Likert scale data obtained from respondents, a predefined guideline was used as a reference to categorize the levels of agreement (Table 1) [27].

**Table 1:** Guidelines for the interpretation of the mean score obtained from the likert scale

Level of agreement	Interpretation value
Strongly disagree	1.00–1.80
Disagree	1.81–2.60
Neutral/uncertain	2.61–3.40
Agree	3.41–4.20
Strongly agree	4.21–5.00

The inferential statistical analysis was done using multiple linear regressions. Hence, the assumptions underlying the linear regressions, including normality, linearity, multi-collinearity, and correlations, were checked before running the final analyses. Normality was measured and checked by P-P plot, Skewness, and Kurtosis: For a sample size < 300, the absolute Z value of skewness and kurtosis should be less than 3.29 [28]. The linearity among pairs of variables was examined by inspections of scatter plots of residuals. Linearity is asserted when the residuals in the plot fall on the diagonal regression line. Multicollinearity among independent variables was checked by using tolerance and VIF (Variance Inflation Factor) with cut points of > 0.2 and < 3, respectively. The inferential statistical analysis was done to determine the association between dependent and independent variables, and a p-value < 0.05 was considered significant. Demographic variables were examined as possible confounders but showed no significant effects, so they were excluded to keep the model concise and focused. Finally, the results were presented using tables and graphs.

### Data quality assurance

The data collection tool was developed in English, and the intended outcomes and content validity were examined. Before the actual data collection, a pre-test was carried out at the EPSS Adama hub on 10% of the main sample size. A reliability test was then undertaken, and adjustments and arrangements were made in response to Cronbach’s alpha values, which varied from 0.72 to 0.83. All constructions had acceptable thresholds, as indicated by Cronbach’s alpha values for the items evaluating internal consistency, ranging from 0.7 to above. To verify the construct validity of the Likert scale data, an Exploratory Factor Analysis using Principal Axis Factoring with a Varimax rotation was conducted. The Kaiser-Meyer-Olkin measure confirmed the sampling adequacy for the analysis, KMO = 0.742, and Bartlett’s test of sphericity was significant, χ² (15) = [195.516], *p* < 0.000, indicating that the correlations between variables were sufficient for Exploratory Factor Analysis. The rotated factor matrix analysis showed all variables loading strongly (> 0.40) on their primary factor and with minimal cross-loadings. This is indicated by the factor 1 (strategic supplier partnership, internal integration practices, and operational performance of the CEPSS and central clusters) and factor 2 (level of information sharing, quality of information sharing, and customer service management) outputs (Table 2). These results support the underlying construct structure of the measurement instruments’ validity.

**Table 2:** KMO and bartlett’s test and rotated factor matrix in CEPSS and central cluster hubs of addis Ababa, Ethiopia, 2024 (*n* = 169)

KMO and Bartlett’s Test										
						Approximate Chi-square		df		sig
Kaiser-Meyer-Olkin					0.742					
Bartlett’s Test of Sphericity						195.516		15		0.000
Rotated Factor Matrix										
		Strategic supplier partnership	Level of information sharing	Quality of information sharing		Customer service management	Internal Integration Practices		Operational performance of CEPSS	
Factor	1	0.530	0.277	0.282		-	0.774		0.522	
	2	-	0.622	0.463		0.667	0.168		0.217	

Extraction Method: Principal Axis Factoring

## Results

### Socio-demographic characteristics of respondents

Of the 175 questionnaires distributed to study participants, 169 were completed and returned, yielding a 96.6% response rate. Of the total participants, 102 (60.35%) were males, and most of the participants were between the ages of 25–30 years. Out of all the participants, 123 (72.78%) had a bachelor’s degree, 81 (43.93%) had less than five years of work experience, and 105 (62.13%) were pharmacists (Table 3).

**Table 3:** Socio-demographic characteristics of the respondents in the CEPSS and the central clusters of addis Ababa, Ethiopia, 2024 (*N* = 169)

Demographic variables		Frequency	Percentage
Age (Years)	Below 25 years	31	18.34
	25–30	63	37.28
	31–35	53	31.36
	36–40	14	8.28
	40 Above years	8	4.73
Gender	Male	102	60.35
	Female	67	39.65
Educational Qualification	Diploma	21	12.43
	Bachelor Degree	123	72.78
	Master’s Degree and above	25	14.79
Type of profession	Pharmacy	105	62.13
	Biomedical engineering	22	13
	Medical laboratory	26	15.38
	Medical doctor	4	2.37
	other	12	7.1
Experiences	Below 5 years	81	47.93
	5–10 years	45	26.62
	11–15 years	30	17.75
	Above 15 years	13	7.69
Working position	Officers	142	84
	Director	19	11.24
	Coordinator	8	4.76

### Strategic supplier partnership

Out of the total respondents, 91 (53.8%) agreed that quality is the primary consideration taken into account by the CEPSS and central cluster when choosing suppliers, with a mean score of 3.34 (Std.Dev = 0.851). Eighty (47.3%) respondents also agreed that planning and goal-setting activities are included in the management of key suppliers in their supply services, with a mean score of 3.36 (Std.dev = 0.895). In terms of how the CEPSS and the central clusters deal with suppliers, 76 (45%) respondents rated in favor of an open and honest approach, while 91 (53.8%) respondents favored relying on a few high-quality suppliers. Additionally, 98 (58%) of the respondents thought that the CEPSS and central clusters are very dedicated to the agreements they have signed; 33 (19.5%) disagreed, with a mean score of 3.17 (Std.Dev = 1.027). The overall mean was 3.38 with a standard deviation of 0.503 (Table 4).

**Table 4:** Strategic supplier partnership management in the CEPSS and the central clusters of addis Ababa, Ethiopia, 2024(*N* = 169)

Description of Strategic Supplier Partnership	Level of agreement					Mean	Std.Dev
	SD (%)	D (%)	*N* (%)	A (%)	SA (%)		
Our firm relies on a few dependable suppliers	4(2.4)	26(15.4)	64(37.9)	75(44.4)	0	3.24	0.798
Our firm considers the quality factor as one of the main criteria in selecting our suppliers	8(4.7)	18(10.7)	52(30.8)	91(53.8)	0	3.34	0.851
Our firm provides any help to improve the quality of suppliers’ products	7(4.1)	16(9.5)	76(45)	64(37.9)	6(3.6)	3.27	0.843
Our firm has continuous improvement programs that include our key suppliers	12(7.1)	15(8.9)	59(34.9)	66(39.1)	17(10.1)	3.36	1.021
Planning and goal-setting activities in our firm are included in our key suppliers	10(5.9)	12(7.1)	61(36.1)	80(47.3)	6(3.6)	3.36	0.895
New product development processes in our firm are included in our key suppliers	19(11.2)	18(10.7)	40(23.7)	76(45)	16(9.5)	3.31	1.139
Our firm certifies our suppliers for quality	12((7.1)	14(8.3)	51(30.2)	74(43.8)	18(10.7)	3.43	1.027
Our suppliers deal with us in an open and honest way	15(8.9)	2(1.2)	55(32.5)	76(45)	21(12.4)	3.51	1.03
Our suppliers have high reliability	20(11.8)	13(7.7)	59(34.9)	73(43.2)	4(2.4)	3.17	1.027
Our firm relies on a few high-quality suppliers	15(8.9)	14(8.3)	46(27.2)	91(53.8)	3(1.8)	3.31	0.977
Our transactions with suppliers have to be closely monitored/supervised	16(9.5)	11(6.5)	26(15.4)	102(60.4)	14(8.3)	3.51	1.058
There is a willingness from our suppliers to provide us with a lot of assistance without exception	15(8.9)	3(1.8)	51(30.2)	83(49.1)	17(10.1)	3.5	1.013
We expect to leverage the business between our suppliers and us in the future	20(11.8)	8(4.7)	43(25.4)	89(52.7)	9(5.3)	3.35	1.007
Our suppliers are highly committed to the agreements signed by us	14(8.3)	15(8.9)	30(17.8)	98(58)	12(7.1)	3.47	1.035
There is a clearer understanding between our suppliers and us about the aims and objectives	7(4.1)	4(2.4)	51(30.2)	101(59.8)	6(3.6)	3.56	0.785
Overall (all items): 15						3.38^(+)^	0.503^(+ )^

SD: Strongly Disagree; D: Disagree; N: Neutral; A: Agree; SA: Strongly Agree; Std.Dev: Standard deviation; ^(+)^ summative

### Level of information sharing

The result indicated that the overall mean was 3.41 with a standard deviation of 0.589. Nearly half of the respondents, 78 (46.2%), agreed that the CEPSS and central clusters share proprietary information with trading partners, yielding a mean value of 3.33 (Std.Dev = 0.835). Eighty-five (50.3%) study participants confirmed their trade partners would fully tell them of any issues that could affect the CEPSS and the central clusters, with a mean score of 3.51 (Std.Dev = 0.880). The CEPSS and central clusters exchanged information with trading partners to build a business strategy valued at an average score of 3.43. Instead, 26 (15.38%) respondents disagreed with the claim that partners would be informed in advance in case of necessary changes (Table 5).

**Table 5:** Level of information sharing in the CEPSS and the central clusters of addis Ababa, Ethiopia, 2024(*N* = 169)

Description Level of information sharing	Level of agreement					Mean	Std.Dev
	SD (%)	D (%)	*N* (%)	A (%)	SA (%)		
In case of any change needed, our partners will be informed in advance	9(5.3)	17(10.1)	54(32)	83(49.1)	6(3.6)	3.36	0.909
Our firm shares proprietary information with trading partners	8(4.7)	13(7.7)	67(39.6)	78(46.2)	3(1.8)	3.33	0.835
If any issues might affect our firm, our trading partners will keep us fully informed	6(3.6)	13(7.7)	52(30.8)	85(50.3)	13(7.7)	3.51	0.88
Our trading partners share firm knowledge to develop our core firm’s processes	11(6.5)	12(7.1)	52(30.8)	86(50.9)	8(4.7)	3.40	0.934
Our firm exchanges information with trading partners to establish our business planning	7(4.1)	16(9.5)	53(31.4)	83(49.1)	10(5.9)	3.43	0.898
Our firm keeps in touch with our trading partners and informs each other about any changes that may affect them.	8(4.7)	17(10.1)	45(26.6)	89(52.7)	10(5.9)	3.45	0.925
Overall (all items): 6						3.41^(+)^	0.589^(+ )^

SD: Strongly Disagree; D: Disagree; N: Neutral; A: Agree; SA: Strongly Agree; Std.Dev: Standard deviation; (+) summative

### Quality of information sharing

The results show that 86 (50.9%) respondents agreed that information exchanged between the CEPSS and central clusters and their partners is accurate (mean score = 3.46, Std.Dev = 1.1180), and 77 (45.6%) respondents agreed that information exchanged between the CEPSS and central clusters and their partners is timely (mean score = 3.58, Std.Dev = 0.955). Likewise, 82 (48.5%) of the participants agreed that information exchange between the CEPSS and the central clusters with their partners is adequate (mean score = 3.52, Std.Dev = 1.097). Of the respondents, 60(35.5%) had no opinion regarding the reliability of the information exchanged between their company and its partners. The overall mean of the quality of information sharing on operational performance of the CEPSS and central clusters was 3.548 with a standard deviation of 0.543 (Table 6).

**Table 6:** Quality of information sharing in the CEPSS and the central clusters of addis Ababa, Ethiopia, 2024 (*N* = 169)

Description of the quality of Information Sharing	Level of agreement					Mean	Std. Dev
	SA (%)	A (%)	*N* (%)	A (%)	SA (%)		
Information exchange between our firm and our partners is timely	8(4.7)	9(5.3)	52(30.8)	77(45.6)	23(13.6)	3.58	0.955
Information exchange between our firm and our partners is accurate	16(9.5)	16(9.5)	31(18.3)	86(50.9)	20(11.8)	3.46	1.118
Information exchange between our firm and our partners is complete	10(5.9)	11(6.5)	38(22.5)	77(45.6)	33(19.5)	3.66	1.052
Information exchange between our firm and our partners is adequate	14(8.3)	14(8.3)	35(20.7)	82(48.5)	24(14.2)	3.52	1.097
Information exchange between our firm and our partners is reliable	15(8.9)	6(3.6)	60(35.5)	63(37.3)	25(14.8)	3.46	1.074
Overall (all items): 5						3.54^(+)^	0.543^(+ )^

SD: Strongly Disagree; D: Disagree; N: Neutral; A: Agree; SA: Strongly Agree; Std.Dev: Standard deviation; (+) summative

### Customer service management

The overall mean score was 3.44, with a standard deviation of 0.48. This means that respondents tended to rank the item above the midpoint of the scale. The relatively modest spread of scores shows that most participants gave comparable assessments, with low variation throughout the group. Also, the results revealed that 84 (49.7%) study participants confirmed that the CEPSS and the central clusters offer and facilitate any support for their customers. In comparison, 89 (52.7%) respondents agreed that their firm frequently interacts with customers to be responsive, reliable, and conform to other standards. Yet, 21 (12.42%) respondents disagreed that CEPSS and the central clusters regularly measure and evaluate customer satisfaction (mean score = 3.50, Std.Dev = 0.881) (Table 7).

**Table 7:** Customer service management in the CEPSS and the central clusters of addis Ababa, Ethiopia, 2024(*N* = 169)

Description: Customer service management	Level of agreement					Mean	Std.Dev
	SD (%)	D (%)	*N* (%)	A (%)	SA (%)		
Our firm frequently interacts with customers to be responsive, reliable, and meet other standards	7(4.1)	3(1.8)	61(36.1)	89(52.7)	9(5.3)	3.53	0.802
Our firm frequently follows and monitors our customers for quality/service feedback	9(5.3)	10(5.9)	66(39.1)	76(45)	8(4.7)	3.44	0.899
Our firm frequently measures and evaluates customer satisfaction	8(4.7)	13(7.7)	54(32)	84(49.7)	10(5.9)	3.5	0.881
Our firm frequently tries to determine future customer expectations	5(3)	11(6.5)	66(39.1)	69(40.8)	18(10.7)	3.41	0.863
Our firm provides and facilitates any assistance for our customers	10(5.9)	6(3.6)	63(37.7)	84(49.7)	6(3.6)	3.38	0.906
Our firm periodically assesses the importance of our relationship with customers	7(4.1)	16(9.5)	63(37.3)	71(42)	12(7.1)	3.63	0.753
Overall (all items): 6						3.44^(+)^	0.476^(+ )^

SD: Strongly Disagree; D: Disagree; N: Neutral; A: Agree; SA: Strongly Agree; Std.Dev: Standard deviation; (+) summative

### Internal integration practices

A significant proportion of respondents, 85 (50.3%), perceived that there is informal cooperation in the organization (mean score = 3.63, Std.Dev = 0.753). Similarly, 81 (47.9%) respondents agreed that the CEPSS and the central clusters facilitate the sharing of ideas, information, and other resources within the organization (mean score = 3.59, Std.Dev = 0.8760). Furthermore, 87 (51.5%) respondents acknowledged the presence of joint planning efforts aimed at anticipating organizational challenges, while 99 (58.6%) affirmed that their firm contributes to resolving operational problems within the organization. Moreover, the overall mean score for internal integration practices in relation to the CEPSS and the central clusters’ operational performance was 3.36, with a standard deviation of 0.48(Table 8).

**Table 8:** Internal integration practices in the CEPSS and the central clusters of addis Ababa, Ethiopia, 2024 (*N* = 169)

Description of Internal Integration Practices	Level of agreement					Mean	Std.Dev
	SD (%)	D (%)	*N* (%)	A (%)	SA (%)		
There is informal teamwork in an organization	2(1.2)	6(3.6)	60(35.5)	85(50.3)	16(9.5)	3.63	0.753
We share ideas, information, and other resources within the organization	5(3)	10(5.9)	54(32)	81(47.9)	19(11.2)	3.59	0.876
There is established teamwork in an organization	2(1.2)	6(3.6)	61(36.1)	84(49.1)	16(9.5)	3.63	0.754
There is joint planning to anticipate and resolve operational problems in the organization	6(3.6)	8(4.7)	48(28.4)	87(51.5)	20(11.8)	3.63	0.884
There is a joint establishment of objectives	8(4.7)	5(3)	37(21.9)	99(58.6)	20(11.8)	3.7	0.892
There is a joint development of responsibilities and understanding in the organization	12(7.1)	3(1.8)	43(25.4)	92(54.4)	19(11.2)	3.61	0.964
There are joint decisions about ways to improve cost efficiencies in the organization	13(7.7)	5(3)	53(31.40	82(48.5)	16(9.5)	3.49	0.983
Overall (all items): 7						3.61^(+)^	0.483^(+ )^

SD: Strongly Disagree; D: Disagree; N: Neutral; A: Agree; SA: Strongly Agree; Std.Dev: Standard deviation; (+) summative

### Operational performance

The CEPSS and the central cluster’s operational performance had an overall mean of 3.47 and a standard deviation of 0.46, which implies that there were relatively moderate differences among respondents. Furthermore, a total of 88 (52.1%) respondents emphasized that the CEPSS and the central clusters are effective in maintaining inventory costs at a minimal level. Also, 70(41.1%) respondents agreed that the CEPSS and the central clusters contribute to minimizing supply service operational costs (mean scores = 3.51, Std.Dev = 0.99). The results further demonstrate that a majority of respondents agreed on the operational performance aspect related to the company’s prompt resolution of customer complaints (mean score 3.53, Std.Dev = 0.94). Moreover, 89 (52.7%) respondents agreed (mean score = 3.43, Std.Dev = 0.88) that the supply service exhibits the capability to adapt swiftly and replenish inventory in response to unforeseen client demands (Table 9).

**Table 9:** Operational performance of EPSS in the CEPSS and the central clusters of addis Ababa, Ethiopia, 2024 (*N* = 169)

Description: Operational performance of EPSS	Level agreement					Mean	Std.Dev
	SD (%)	D (%)	*N* (%)	A (%)	SA (%)		
The supply service logistics costs are kept at a minimum level	13(7.7)	5(3)	53(31.4)	82(48.5)	16(9.5)	3.53	0.893
The supply service inventory costs are kept at a minimum level	10(5.9)	12(7.1)	42(24.9)	88(52.1)	17(10.1)	3.53	0.976
The supply service operation costs are kept at a minimum level	9(5.3)	11(6.5)	56(33.1)	70(41.1)	23(13.6)	3.51	0.989
We provide a better quality of service	13(7.7)	13(7.7)	46(27.2)	70(41.1)	27(16)	3.5	1.092
Our supplier’s pharmaceuticals have good quality	10(5.9)	15(8.9)	53(31.4)	74(43.8)	17(10.1)	3.43	0.992
Product loss on arrival is very low	12(7.1)	9(5.3)	44(26)	77(45.6)	27(16)	3.58	1.050
The supply service has an excellent on-time delivery record to customers.	10(5.9)	12(7.1)	47(27.8)	83(49.1)	17(10.1)	3.5	0.977
Our partners’ deliveries are reliable and accurate	4(2.4)	16(9.5)	52(30.8)	83(49.1)	14(8.3)	3.51	0.867
Our company solves customer complaints promptly	8(4.7)	13(7.7)	46(27.2)	86(50.9)	16(9.5)	3.53	0.939
The supply service can quickly introduce new products into the market with the existing system	10(5.9)	15(8.9)	60(35.5)	76(45)	8(4.7)	3.34	0.925
The supply service can quickly adjust and refill unexpected needs from customers	10(5.9)	8(4.7)	56(33.1)	89(52.7)	6(3.6)	3.43	0.878
Our supply service can supply different features of products, such as options, sizes, and generics	11(6.5)	16(9.5)	74(43.8)	60(35.5)	8(4.7)	3.22	0.924
Overall (all items): 12						3.47^(+)^	0.462^(+ )^

SD: Strongly Disagree; D: Disagree; N: Neutral; A: Agree; SA: Strongly Agree; Std.Dev: Standard deviation; (+) summative

### Composite constructs of study variables

Each construct is represented by a group of items, and the mean scores show how strongly participants agreed with statements in each part. Operational performance, measured with twelve items, with a mean of 3.47. This suggests that staff generally see performance as moderately strong, with responses clustering fairly around that average. Strategic supplier partnership has the lowest mean among the constructs at 3.38 across fifteen items. The means float around the mid-three range, pointing to moderate perceptions across all domains, with integration practices and information quality standing out slightly above the rest.

### Multiple regression analysis

Before interpreting the regression analysis, the underlying assumptions of the multiple regression were rigorously evaluated.

### Multiple linear regression assumptions

Before interpreting the regression analysis, all the assumptions of the multiple regression should be fulfilled to get a reliable and dependent result of the analysis. Accordingly, normality test, linearity test, and multicollinearity test were performed.

#### Normality test

**Fig. 2:**
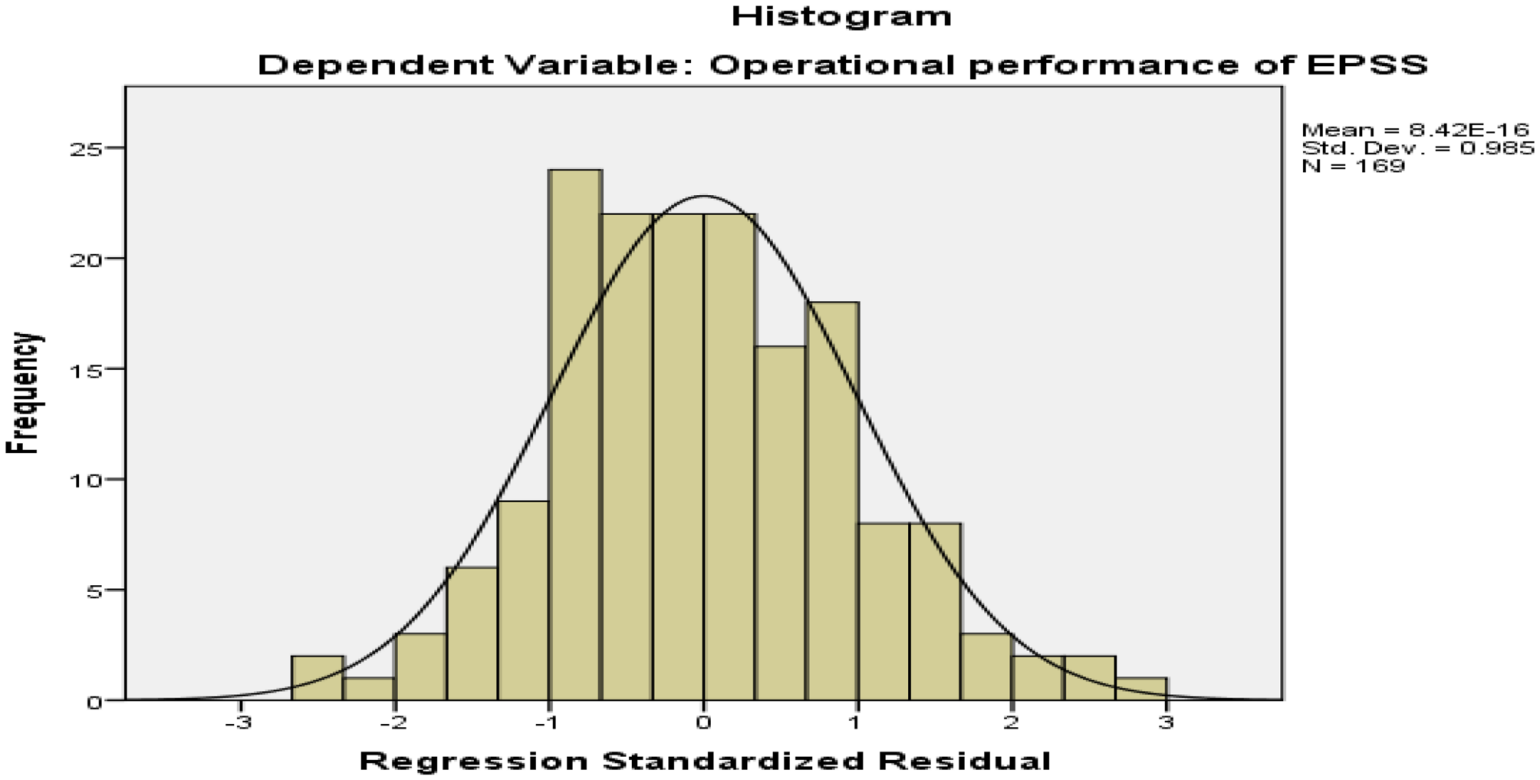
Histogram on a normal distribution of residuals against the predicted dependent variable scores in the CEPSS and the central clusters of Addis Ababa, Ethiopia, 2024 (*n* = 169)

A visual examination of the histogram showed a normal distribution of residuals against the predicted dependent variable scores. The absolute value of skewness and kurtosis of the data was tested and found within the accepted threshold value, Z < 3.29 (Fig. 2; Table 10).

**Table 10:** Skewness and kurtosis values for normality test in the CEPSS and central clusters of addis Ababa, Ethiopia, 2024 (*n* = 169)

Parameters	Strategic supplier partnership	Level of information sharing	Quality of information sharing	Customer service management	Internal Integration Practices	Operational performance
Skewness	− 0.413	− 0.197	-0.07	− 0.426	0.602	0.399
Std. Error of Skewness	0.187	0.187	0.187	0.187	0.187	0.187
Kurtosis	− 0.179	0.121	0.782	0.589	0.521	− 0.101
Std. Error of Kurtosis	0.371	0.371	0.371	0.371	0.371	0.371

### Linearity test

#### Homoscedastic test

**Fig. 3:**
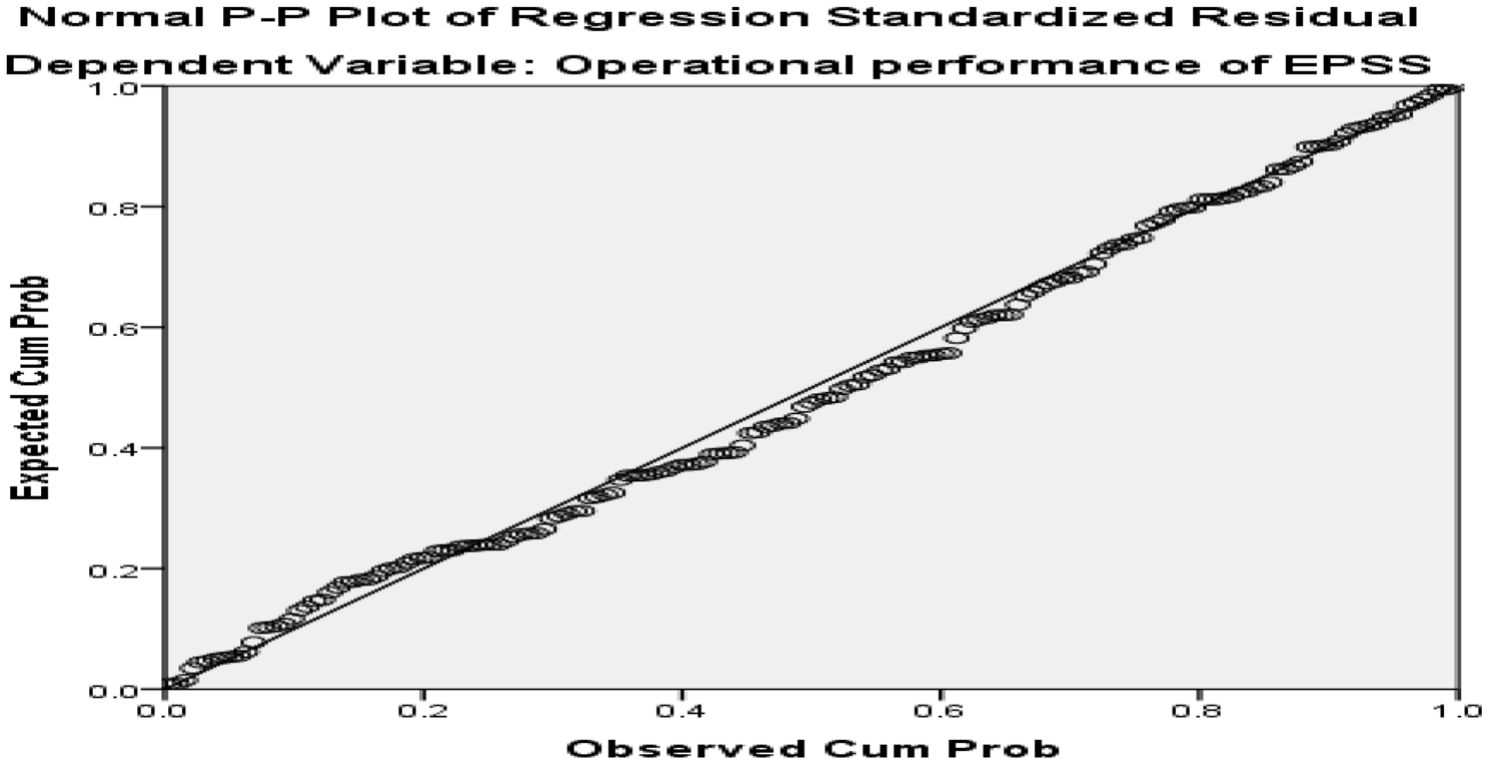
P-P Plot of standardized regression standardized residual in the CEPSS and central clusters of Addis Ababa, Ethiopia, 2024 (*n* = 169)

**Fig. 4:**
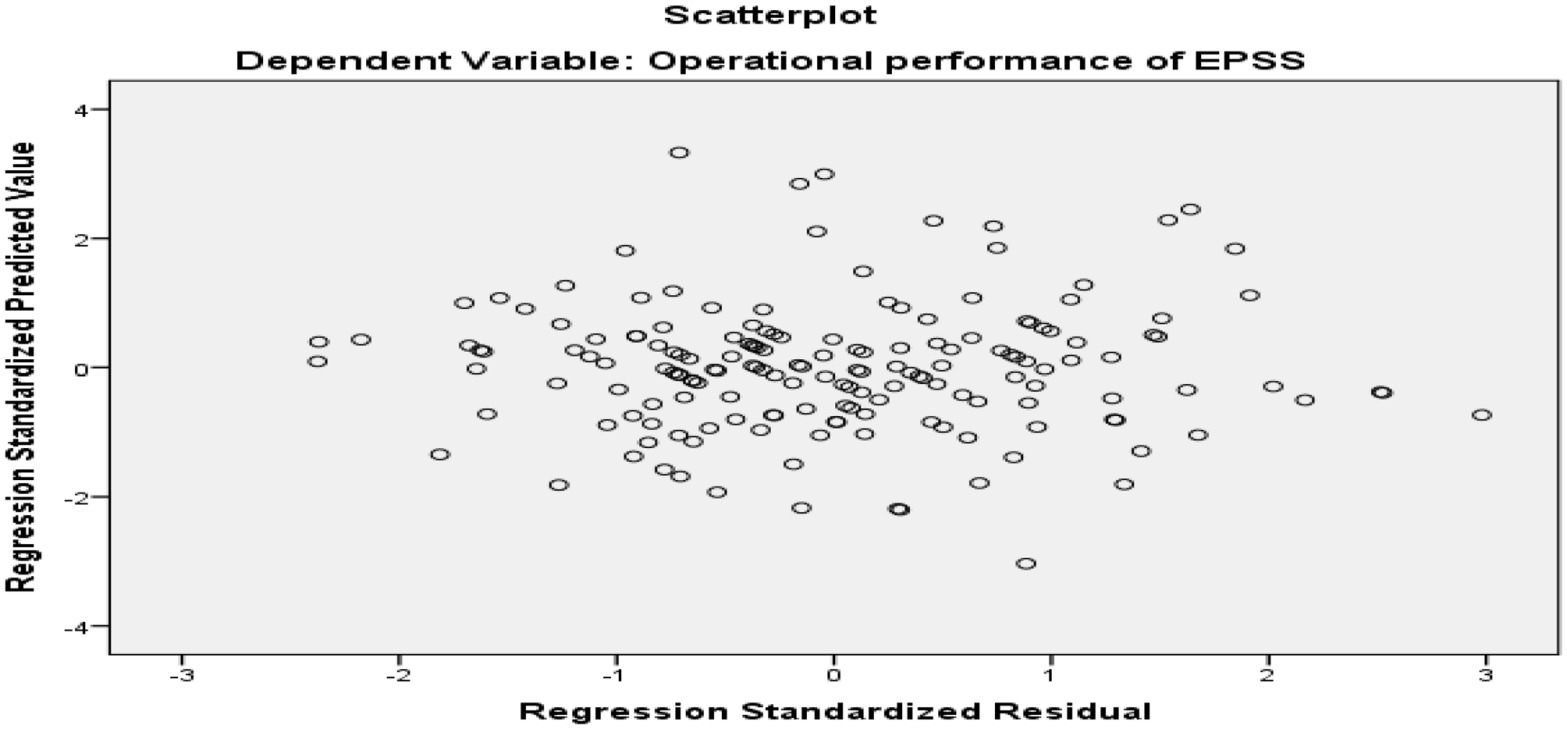
Scatter plot based on residual and predictive value in the CEPSS and central clusters of Addis Ababa, Ethiopia, 2024 (*n* = 169)

To assess linearity, scatter plots of residuals were sketched and inspected. The scatter plots demonstrate a linear relationship between the variables. Homoscedasticity can be evaluated by visually examining a plot of the standardized residuals plotted against the standardized predicted values from the regression. Additionally, the residuals output has a sound normal distribution because the plotted residuals were around the diagonal straight line (Figs. 3 and 4).

### Multicollinearity

This assumption can be evaluated by examining the tolerance and the variance inflation factor (VIF). A VIF value below 10 and tolerance statistics above 0.2 indicate the absence of multicollinearity within the data. Based on the data presented, the VIF is less than 2, and the tolerance exceeds 0.2. Consequently, it can be concluded that there is no multicollinearity present in the regression model. In addition, the value of the correlation coefficient (r) < 1 for all bivariate correlations among independent variables, which indicates there is no multicollinearity problem in the model (Tables 11 and 12**)**.

**Table 11:** The result of the multicollinearity test for independent variables in the CEPSS and central clusters of addis Ababa, Ethiopia, 2024 (*n* = 169)

Variables	Collinearity Statistics		95.0% Confidence Interval of B	
	Tolerance	VIF	Lower Bound	Upper Bound
Constant			0.151	1.412
Strategic supplier partnership	0.804	1.244	0.039	0.297
Level of information sharing	0.718	1.393	0.048	0.282
Quality of information sharing	0.777	1.286	0.004	0.248
Customer service management	0.775	1.290	− 0.051	0.227
Internal Integration Practices	0.695	1.440	0.079	0.369

**Table 12:** Bivariate correlation among the independent and dependent variables in the CEPSS and central clusters of addis Ababa, Ethiopia, 2024 (*n* = 169)

	OP	SSP	LIS	QIS	CSM	IIP
OP	1					
SSP	0.354*	1				
LIS	0.413*	0.196	1			
QIS	0.353*	0.123	0.349*	1		
CSM	0.174	0.115	0.426*	0.320*	1	
IIP	0.442*	0.433*	0.326*	0.342*	0.108	1

OP: Operational performance of CEPSS, SSP: Strategic supplier partnership, LIS: Level of information sharing, QIS: Quality of information sharing, CSM: Customer service management, IIP: Internal Integration practices. *: Correlation is significant at the 0.05 level (2-tailed)

### Model summary

The regression model demonstrates a moderate to strong positive linear relationship (*R* = 0.576) between the predictors and the outcome, with a statistically significant relationship, explaining approximately 33.2% of the variance in the outcome. The low standard error of the estimate (0.38324) suggests that the model provides a reasonably good fit to the data, where the model’s predictions are relatively close to the actual values. The significant F-change (*p* < 0.001) confirms that the inclusion of the predictors improves the model’s explanatory power (Table 13).

**Table 13:** Model summary statistics for assessing goodness-of-fit

Model Summary									
Model	*R*	*R* Square	Adjusted *R* Square	Std. Error of the Estimate	Change Statistics				
					*R* Square Change	F Change	df1	df2	Sig. F Change
	.576^a^	0.332	0.312	0.38324	0.332	16.226	5	163	0.000

a. Predictors: (Constant), Internal Integration practices, Customer service management, Strategic supplier partnership, Quality of information sharing, Level of information sharing

### Analysis of ANOVA

The ANOVA result revealed that the F-statistic is 16.226, and the associated p-value is highly significant (*p* < 0.050). This indicates that the regression model as a whole is statistically significant. This implies that the regression model has a less than 0.01 likelihood of giving a wrong prediction. Therefore, it is concluded that the regression model significantly predicts the outcome variable (Table 14).

**Table 14:** Significance level for multiple correlation coefficient-ANOVA

ANOVA^a^					
Model	Sum of Squares	Df	Mean Square	F	Sig.
Regression	11.916	5	2.383	16.226	.000^b^
Residual	23.940	163	0.147		
Total	35.856	168			

a. Dependent Variable: Operational performance of the CEPSS and the central clusters

b. Predictors: (Constant), Internal Integration practices, Customer service management, Strategic supplier partnership, Quality of information sharing, Level of information sharing

### Multiple linear regression coefficients

A multiple linear regression analysis was performed to examine the predictive relationship between the independent variables and the dependent variable, yielding the following multiple regression equation:

$$\:Y\:0\hspace{0.17em}+\hspace{0.17em}1X1\hspace{0.17em}+\hspace{0.17em}2X2\hspace{0.17em}+\hspace{0.17em}3X3\hspace{0.17em}+\hspace{0.17em}4X4\hspace{0.17em}+\hspace{0.17em}5X5\hspace{0.17em}+\hspace{0.17em}\epsilon$$ 

Where: Y operational performance of the EPSS.

β0 = the intercept (the value of Y when x1, x2, x3, x4 and x5are zero).

β1 = Regression coefficient for Strategic supplier partnership (x1).

β2 = Regression coefficient for Level of information sharing (x2).

β3 = Regression coefficient for Quality of information sharing (x3).

β4 = Regression coefficient for Customer service management (x4).

β5 = Regression coefficient for Internal Integration practices (x5).

ε = error term.

The final result presented by the model is as follows: Y = 0.782 + 0.168 × 1 + 0.165 × 2 + 0.126 × 3 + 0.088 × 4 + 0.224 × 5 + ε (Table 15). The regression analysis revealed that strategic supplier partnership (β = 0.168, t = 2.564, *p* < 0.010), level of information sharing (β = 0.165, t = 2.782, *p* < 0.006), quality of information sharing (β = 0.126, t = 2.045, *p* < 0.042 and internal integration practices (β = 0.224, t = 3.046, *p* < 0.030) have a positive effect on the operational performance of CEPSS and the central cluster. Holding all other variables constant, a unit increase in strategic supplier partnership leads to a 16.8% increase in the operational performance of CEPSS and the central cluster. Similarly, keeping all other variables constant, a unit increase in the level of information results in a 16.5% increase in the operational performance of the supply service (Table 16).

**Table 15:** Descriptive statistics for summative constructs of study variables in the CEPSS and the central clusters of addis Ababa, Ethiopia, 2024 (*n* = 169)

Construct	Number of Items	Mean ( summative)	Standard Deviation(summative)
Operational performance	12	3.47	0.462
Strategic supplier partnership	15	3.38	0.503
Level of information sharing	6	3.41	0.589
Quality of information sharing	5	3.54	0.543
Customer service management	6	3.44	0.476
Inter Integration Practices	7	3.61	0.483

**Table 16:** Regression coefficients for the association between supply chain management practice and supply chain performance of the CEPSS and the central clusters of addis Ababa, Ethiopia, 2024 (*N* = 169)

Model	Unstandardized Coefficients		Standardized Coefficients	t	Sig.
	B	Std. Error	Beta		
(Constant)	0.782	0.319		2.447	0.015
Strategic supplier partnership	0.168	0.065	0.183	2.564	0.011
Level of information sharing	0.165	0.059	0.210	2.782	0.006
Quality of information sharing	0.126	0.062	0.148	2.045	0.042
Customer service management	0.088	0.071	0.091	1.249	0.214
Internal Integration Practices	0.224	0.073	0.234	3.046	0.003

## Discussion

The study’s results were thoroughly explored in this section by making comparisons to other study results from around the world. The study attempted to assess the effect of pharmaceutical supply chain management practice on the operational performance of the CEPSS and the central clusters. The ultimate goal of the pharmaceutical supply service organization is to ensure pharmaceutical accessibility to the end users [29]. The findings of this study showed that the performance of supplier quality is the primary concern of the CEPSS and the central clusters. 47% of the respondents agreed that planning and goal-setting activities are included in the management of key suppliers in their supply services. Correspondingly, the CEPSS and the central clusters deal with suppliers in an open and honest approach, and 58% of the CEPSS and the central clusters’ suppliers are very dedicated to the agreements they have signed. A significant proportion (46.2%) of respondents agreed that the CEPSS and the central clusters share proprietary information with trading partners, and half of its trade partners would fully tell them of any issues that could affect CEPSS and the central clusters. This disagrees with a previous study in which 33.7% of the respondents disagreed that information sharing within the agency is simplified to improve service quality [30]. Half of the respondents agreed that information exchanged between the CEPSS and the central clusters and their partners is accurate, timely, and adequate, which could improve the delivery performance of a supplier. A similar study shows that timely and reliable delivery will improve delivery performance [31]. Regarding customer service management, 52.7% of respondents agreed that the CEPSS and the central clusters frequently interact with customers to be responsive, reliable, and conform to other standards, whereas 12.42% of respondents disagreed that the CEPSS and the central clusters regularly measure and evaluate customer satisfaction. The lack of perceived satisfaction measurement suggests a gap in data-driven service improvements. However, customer service management did not show statistical significance in our model, and therefore cannot be presented as a confirmed predictor.

Regarding internal integration practice, 47.9% of respondents agreed that the CEPSS and the central clusters facilitate the sharing of ideas, information, and other resources within the organization, and more than half of the respondents acknowledged the presence of joint planning efforts aimed at anticipating organizational challenges, which is in line with a previous study [22]. Internal integration among functional areas reduces the order cycle, improves communication, contributes to innovative project development, and improves customer service. On top of this, 52.1% of respondents underlined that the CEPSS and the central clusters are effective in maintaining inventory costs at a minimal level, and contribute to minimizing supply service operational costs and operational performance aspects related to the company’s prompt resolution of customer complaints. However, a previous study conducted in Ethiopia with 343 respondents indicated that 41% of respondents gave a neutral response to the statement that the logistics, inventory, and operating costs were kept to a minimum [22]. The difference in findings between the two studies could be attributed to variations in their sample sizes. The study with a larger sample size may have captured a broader range of participant perspectives, leading to more diverse opinions. In contrast, the current study with a relatively smaller sample might not have fully represented differing viewpoints, potentially skewing the results. Moreover, 52.7% of respondents agreed that the supply service exhibits the capability to adapt swiftly and replenish inventory in response to unforeseen client demands.

The regression analysis of the current study revealed that the level of information sharing and supplier relationship management have significant positive effects on operational performance of pharmaceutical supply services with β = 0.183, *p* = 0.011, and β = 0.210, *p* = 0.006, respectively. Hence, building cooperation with strategic supplier relationships and customers improves inter- and intra-organizational relationships based on a collaborative approach, and this improves the operational performance of a supplier [32]. This study is in line with a similar study that determined a positive and direct relationship between strategic supplier partnership and supply chain performance [30]. The information shared between the CEPSS and the central clusters with their partners is found to be accurate, timely, and adequate, with a mean score of 3.536. Most importantly, the quality of information sharing has a significant positive effect on supply service operational performance [33], which is in agreement with our study that quality of information sharing also has a significant positive effect on supply service operational performance with β = 0.148 at *p* < 0.050. Consequently, the quality of information improves the operational performance of suppliers as it has a positive and direct relationship with information sharing and supply chain performance [34]. On the other hand, the present study indicates that customer service management has a positive but non-significant effect on operational performance (β = 0.088, *p* = 0.214). This suggests that while customer service practices may contribute to operational outcomes, their impact may be moderated by other factors or require further investigation to establish statistical significance. In contrast, prior research highlights that customer relationship management exerts a substantially stronger influence, accounting for the highest variance in performance outcomes (β = 0.78, *p* < 0.001). This underscores the critical role of customer relationship management in enhancing supply chain performance, emphasizing that strategic investments in relationship-building initiatives can lead to significant operational improvements [32]. The other key finding of this study is the significant positive influence of internal integration practices on operational performance (β = 0.234, *p* = 0.003), highlighting their critical role in enhancing organizational efficiency and effectiveness. This result underscores the importance of fostering strong cross-functional collaboration and alignment within firms to achieve superior operational outcomes. As a result, customer and internal integration improves operational performance through a customer-focused approach and long-term relationships, encouraging inter-organizational integration based on collaborative work between departments and arranging internal and external meetings and supply chains [30, 31].

### Limitations and strengths of the study

This contributes to the broader discourse on SCM performance, and has methodological strengths, such as the complete census of all supply-chain practitioners in the CEPSS and the central clusters, the use of validated instruments with high reliability, and the rigorous analytical approach, which included factor analysis and checks on all regression assumptions. However, the limitations should be acknowledged. First, the study’s sample was limited to SCM-active employees within the CEPSS and the central clusters, excluding non-SCM staff and regional hubs, which may limit the generalizability of findings across the entire organization. Second, reliance on self-reported survey data may introduce response biases, suggesting the need for mixed-method approaches that could deepen understanding of SCM dynamics. Third, contextual factors such as infrastructural constraints and technological disparities likely mediate the operational performance of the study settings, but were not explicitly analyzed.

### Practical implications and recommendations

The study provides feasible strategies for enhancing Ethiopia’s supply chain operations for pharmaceuticals. Strengthening strategic supplier collaboration through focused and consistent engagement can reduce delays and improve product quality. Timely, accurate, and consistent information exchange facilitates forecasting, decision-making, and coordination in distribution, warehousing, and procurement. Internal integration, particularly cross-functional collaboration across procurement, logistics, finance, ICT, and customer service, highly predicts operational performance by reducing cycle times and costs. Although there was no statistically significant impact of customer service management, service gaps and bottlenecks were found by systematic feedback gathering from healthcare facilities. Finally, we recommend further study that incorporates more clusters, incorporates a mixed-methods approach to address more complex operational dynamics, and explores the role of infrastructure and technology constraints as potential mediating or moderating variables.

## Conclusion

The current study demonstrates that pharmaceutical supply chain management practices significantly influence the operational performance of the CEPSS and the central clusters. Thus, strategic supplier partnerships, quality of information sharing, internal integration practice, and level of information sharing significantly improve operational performance, collectively accounting for 33.2% of performance variability, underscoring their pivotal role in enhancing the CEPSS and the central clusters functionality. In this regard, internal integration was identified as the strongest contributor to performance, showing its role in enhancing efficiency across the suppliers. Increased cross-department collaboration and better information flow can boost responsiveness, save costs, and improve service reliability. Moreover, customer service management had a favorable but insignificant impact.

## Data Availability

The datasets used in this study were included in the main manuscript.
